# Transesophageal echocardiographic imaging of the coronary sinus: a retrospective analysis of mid-esophageal views and a novel transgastric view

**DOI:** 10.1186/s12871-022-01873-5

**Published:** 2022-10-24

**Authors:** Tzonghuei Chen, Geoffrey Hayward, Patricia Apruzzese, Andrew Maslow

**Affiliations:** 1 Department of Anesthesiology, Warren Alpert Medical School of Brown University, Rhode Island Hospital / Lifespan, 593 Eddy Street, Providence, RI 02903 USA; 2 East Greenwich, USA

**Keywords:** Coronary sinus, Retrograde cardioplegia, Transesophageal echocardiography

## Abstract

**Background:**

Transesophageal echocardiographic imaging plays an important role in assessing coronary sinus anatomy prior to placement of a retrograde cardioplegia cannula. The coronary sinus can be imaged in the long axis by advancing the TEE probe from the mid-esophageal 4-chamber view or using a modified mid-esophageal bicaval view, while a short axis view can be obtained in the mid-esophageal 2-chamber view. While use of a transgastric view is only briefly mentioned in the literature as an alternative to mid-esophageal views, the authors commonly include it in our comprehensive transesophageal echocardiographic exam of the coronary sinus. This study examines the various imaging strategies. We hypothesize that the transgastric view offers comparable coronary sinus imaging to the mid-esophageal views.

**Methods:**

After approval by our institutional review board, the intraoperative transesophageal echocardiographic exams for 50 consecutive elective cardiac surgical patients with a comprehensive echocardiographic assessment of the coronary sinus were retrospectively reviewed and analyzed to evaluate imaging of the coronary sinus in the various views. For each view, we noted and recorded if the coronary sinus and coronary sinus cannula were visualized. Statistical analysis required pairwise comparisons between each of the 4 views. *P* values were calculated using McNemar’s Exact test.

**Results:**

Both the coronary sinus and coronary sinus cannula were visualized a majority of the time for each view. There was no statistically significant difference between each view in its ability to visualize the coronary sinus, nor was there a statistically significant difference between each view in its ability to visualize the coronary sinus cannula.

**Conclusions:**

Use of a transgastric window provides the echocardiographer with an effective alternate modality for imaging the coronary sinus when mid-esophageal views are limited.

## Background

Cannulation of the coronary sinus (CS) is performed to administer retrograde cardioplegia for cardiac surgeries. Whether performed by the surgeon via median sternotomy or percutaneously by an anesthesiologist, transesophageal echocardiographic (TEE) imaging plays an important role in assessing CS anatomy and then guiding and confirming placement of the CS cardioplegia cannula [1–7].

**Fig. 1:**
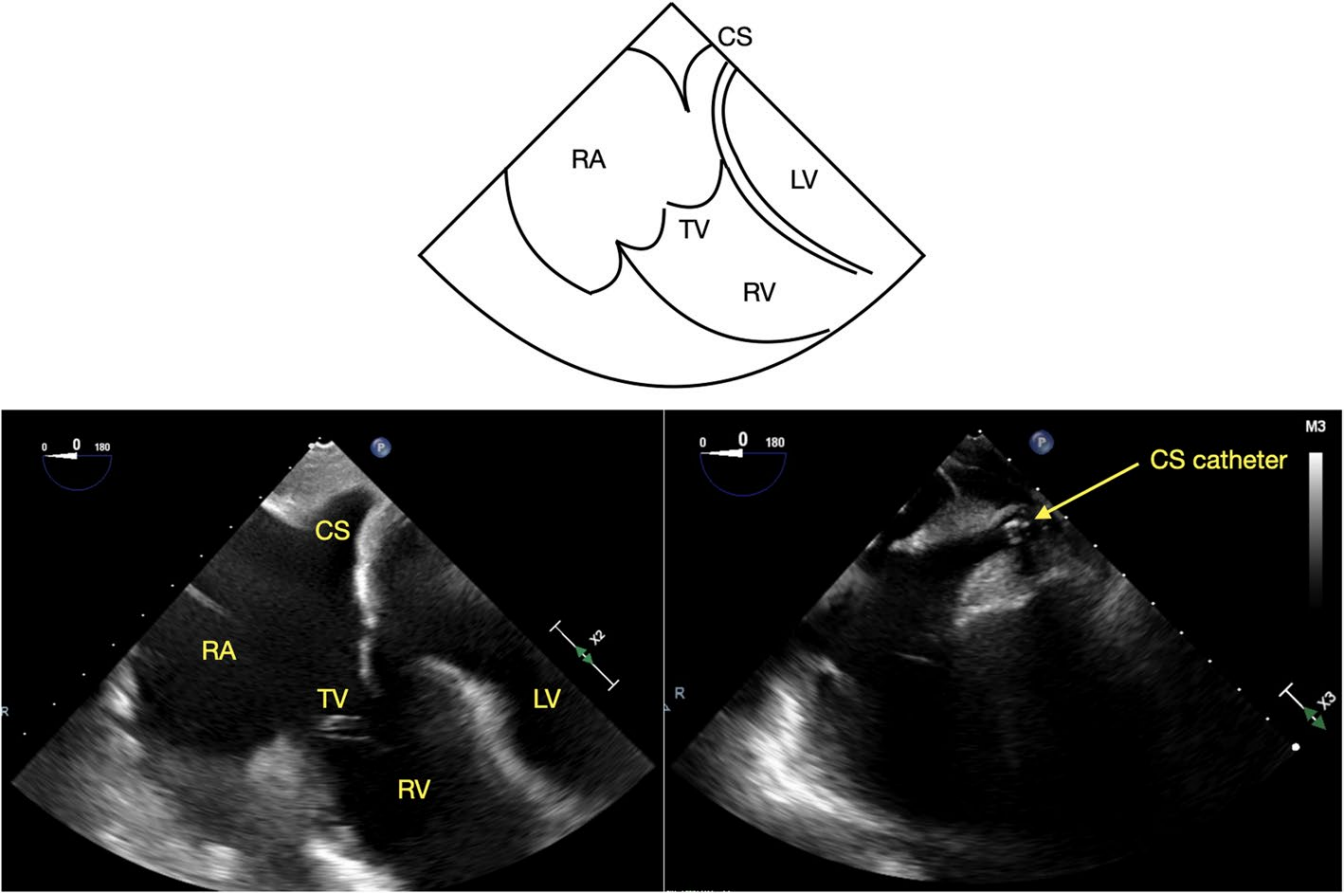
ME 4C view of the coronary sinus with and without a coronary sinus catheter, CS = coronary sinus, RA = right atrium, TV = tricuspid valve, RV = right ventricle, LV = left ventricle

**Fig. 2:**
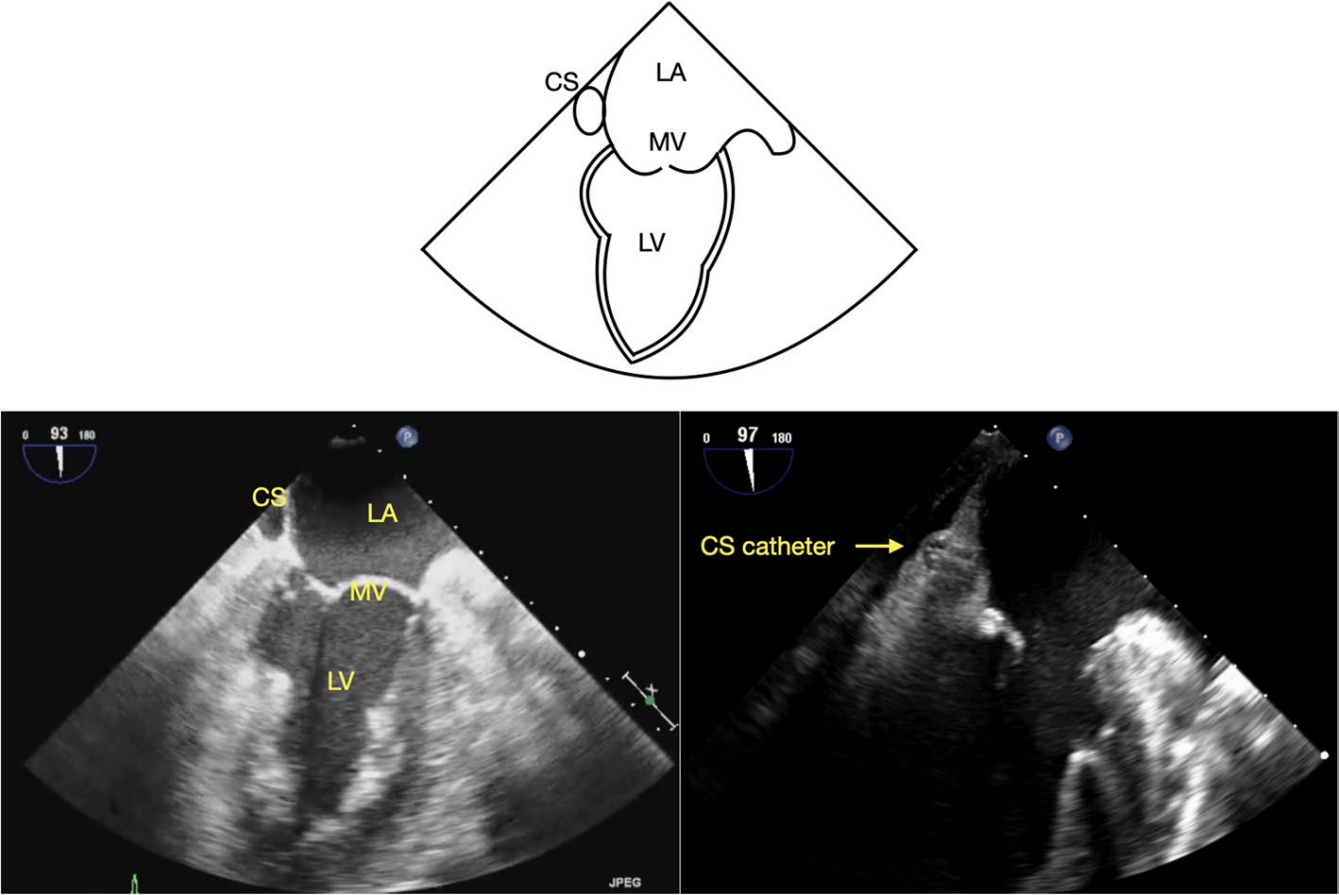
ME 2C view of the coronary sinus with and without a coronary sinus catheter. CS = coronary sinus, LA = left atrium, MV = mitral valve, LV = left ventricle

**Fig. 3:**
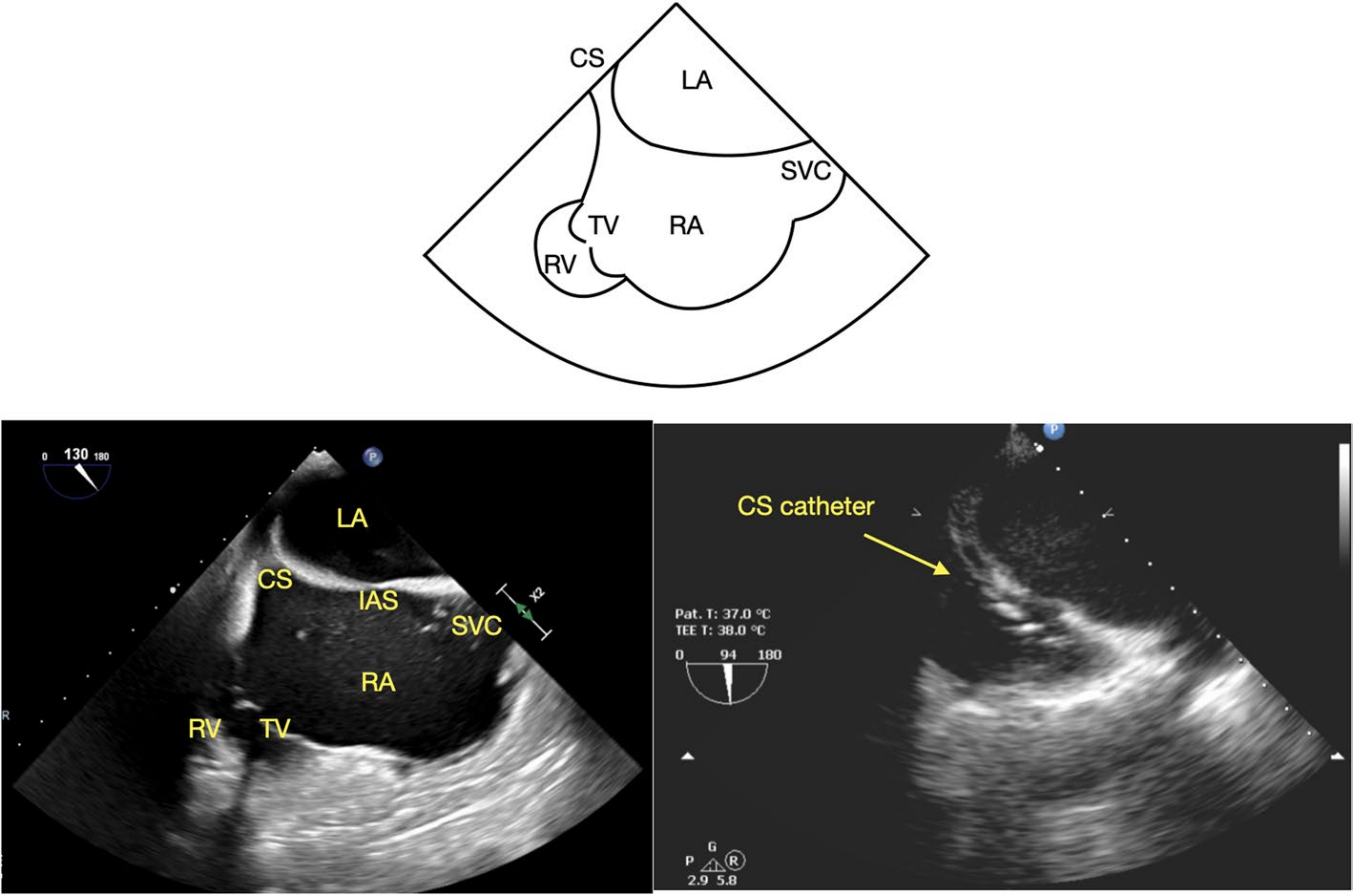
Modified bicaval view of the coronary sinus with and without a coronary sinus catheter. CS = coronary sinus, RA = right atrium, LA = left atrium, IAS = interatrial septum, SVC = superior vena cava, TV = tricuspid valve, RV = right ventricle

The CS can be imaged from mid- and lower-esophageal windows. A long axis view of the CS can be obtained by advancing the TEE probe from the mid-esophageal (ME) 4-chamber view (ME4C) (Fig. 1) [8–10], while a short axis view can be obtained in the ME 2-chamber view (ME2C) (Fig. 2) [8, 9]. The CS can also be visualized in the long axis using a modified ME bicaval view (110–130 degrees) (Fig. 3) [4–6, 9, 10].

**Fig. 4:**
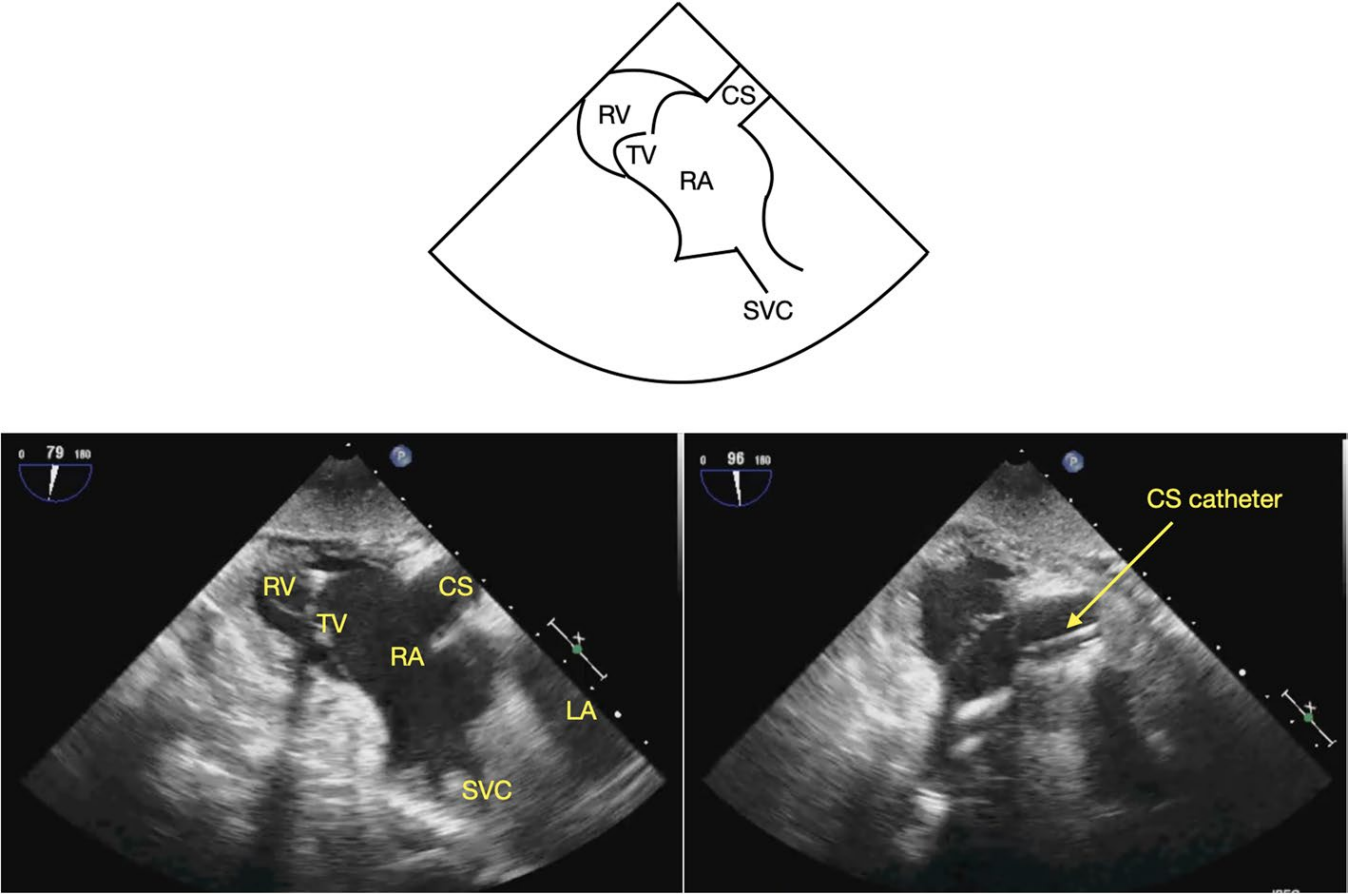
Transgastric view of the coronary sinus with and without a coronary sinus catheter. CS = coronary sinus, RA = right atrium, LA = left atrium, SVC = superior vena cava, IVC = inferior vena cava, TV = tricuspid valve, RV = right ventricle, Ao = aorta, PA = pulmonary artery

This study examines the various CS imaging strategies, including a transgastric view (TG) of the CS (TG-CS) [Fig. 4]. Though this TG-CS view is only briefly mentioned in the literature [9] as an alternative to ME views, the authors commonly include it in our comprehensive TEE assessment of the CS. We hypothesize that the TG-CS view offers comparable CS imaging to the ME views.

## Methods

After approval by our institutional review board, the intraoperative echocardiographic exams for 50 consecutive elective cardiac surgical patients with a comprehensive TEE assessment of the coronary sinus were retrospectively reviewed and analyzed to evaluate imaging of the CS.

Our institutional standard for TEE imaging of the CS includes four standard views. Starting from the ME4C view, slight probe advancement reveals the CS in its long axis just above the attachment of the tricuspid valve (TV) septal leaflet to the interventricular septum (Fig. 1) [8–10]. In the ME2C view, the CS is seen in its short axis immediately above the basal inferior left ventricular (LV) segment (Fig. 2) [8, 9]. The ME modified bicaval view visualizes the CS in its long axis with its ostium adjacent to the TV septal leaflet (Fig. 3) [4–6, 9, 10]. Finally, from the TG LV long axis window (TG LV LAX) with an omniplane angle of 80–100 degrees, the TEE probe is rotated slightly to the right to obtain the TG-CS view (Fig. 4).

All patients received a balanced general endotracheal anesthetic with propofol, rocuronium, and isoflurane and standard non-invasive American Society of Anesthesiology monitoring. Additional invasive monitoring included arterial and central venous catheters. The use of a pulmonary artery catheter as well as patient specific hemodynamic management and medication dosing were left to the discretion of the attending anesthesiologist and conformed to institutional protocols.

All TEE exams were performed after the induction of general anesthesia, prior to cardiopulmonary bypass during stable hemodynamic periods. They were performed with either a Philips Epiq ultrasound system with a X7-2 T probe (Philips Medical Systems, Andover, MA), a Philips IE33 ultrasound system with a X7-2 T probe (Philips Medical Systems, Andover, MA), or a GE Vivid E95 with a multiplane probe (GE, Chicago, IL).

The data was collected and analyzed by two experienced and certified (National Board of Echocardiography) echocardiographers (THC, AM). Specific attention was directed towards imaging of the CS in the ME4C, ME2C, modified ME bicaval, and TG-CS views (Figs. 1, 2, 3 and 4). Views of the CS in the ME4C, ME2C, and modified ME bicaval views were achieved by following available guidelines [4–6, 8–10]. The TG-CS view is achieved by obtaining a TG LV LAX view with the omniplane angle at 80–100 degrees and turning the probe slightly to the right.

For each view, the following was noted and recorded:

1. Was the CS visualized?
2. Was the CS cannula, placed either percutaneously by the anesthesiologist or via median sternotomy by the surgeon, visualized following placement?

Statistical analysis required pairwise comparisons between each of the 4 views. *P* values were calculated using McNemar’s Exact test for the binary variables “CS visible? Yes or No” and “CS cannula visible? Yes or No”.

## Results

The surgeries that the study patients underwent included coronary artery bypass grafting with or without valve repair or replacement, valve repair or replacement, pericardiectomy, and mediastinal exploration and are described in Table 1.

**Table 1:** Surgical procedures performed

	# of patients
CABG	32
CABG + AV replacement	2
CABG + MV repair	1
AV replacement	7
MV repair/replacement	3
TV repair	1
MV repair + TV repair	1
Pericardiectomy	1
Mediastinal exploration	2

*CABG* Coronary artery bypass grafting, *AV* Aortic valve, *MV* Mitral valve, *TV* Tricuspid valve

A comparative analysis of the four views of the CS used in this study is summarized in Table 2. The CS was visible in at least 2 views for every patient and was visible in all 4 views in 45 patients (90%).

**Table 2:** Descriptive statistics and comparative analysis of the four views of the coronary sinus (CS) used in this study. Data are presented as occurrence (%)

	ME4C	ME2C	ME Modified Bicaval	TG-CS
CS visible?				
No	2% (1/50)	4% (2/50)	6% (3/50)	2% (1/50)
Yes	98% (49/50)	96% (48/50)	94% (47/50)	98% (49/50)
CS cannula visible?				
No	0% (29/29)	6.9% (2/29)	13.8% (4/29)	6.9% (2/29)
Yes	100% (29/29)	93.1% (27/29)	86.2% (25/29)	93.1% (27/29)

*ME4C* Mid-esophageal 4 chamber view, *ME2C* Mid-esophageal 2 chamber view, *TG-CS* Transgastric coronary sinus view

The CS was visible 98% of the time from the ME4C view (49/50 patients), 96% of the time from the ME2C view (48/50 patients), 94% of the time from the modified bicaval view (47/50 patients), and 98% of the time from the TG-CS view (49/50 patients).

For the 29 exams which attempted to image the CS cannula after placement, the cannula was visualized 100% (29/29 patients) of the time in the ME4C view, 93% (27/29 patients) of the time in the ME2C view, 86% (25/29 patients) of the time in the modified bicaval view, and 93% (27/29 patients) of the time in the TG-CS view.

There was not a statistically significant difference between each view in its ability to visualize the CS, nor was there a statistically significant difference between each view in its ability to visualize the CS cannula.

## Discussion

The CS is responsible for most of the venous drainage of the heart, receiving coronary venous blood from a number of tributaries including the small, middle, great, and oblique cardiac veins as well as the left marginal vein and the left posterior vein. It travels along the posterior atrioventricular groove of the heart and empties into the right atrium between the inferior vena cava inlet and the septal leaflet of the TV.

Retrograde cardioplegia alone or as an adjunct to antegrade cardioplegia is commonly used in cardiac surgery. Administration of retrograde cardioplegia in patients with coronary artery disease improves cooling of the myocardium distal to the coronary obstructions and may confer long-term benefits for LV systolic function [11]. In patients with aortic insufficiency, retrograde cardioplegia is important to provide adequate myocardial protection without causing LV distention [12]. Cannulation of the CS for retrograde cardioplegia delivery is also critical in minimally invasive cardiac surgery, where the CS cannula is placed percutaneously by the anesthesiologist using TEE guidance [1–7].

Although insertion of a CS cannula can be facilitated and confirmed using fluoroscopic guidance and pressure waveform analysis, perioperative real-time TEE has the benefit of providing continuous visualization of both the CS and CS cannula without the need for contrast or radiation exposure [7] and can both reduce the risk of CS injury [13] and detect complications related to cannulation [14, 15]. Prior to instrumentation of the CS, imaging allows for the identification of anatomic variants that may preclude the use of retrograde cardioplegia such as a persistent left superior vena cava, Thebesian valve [16, 17], atrial septal defect or sinus venosus defect [18], Chiari network [2], or anomalous great cardiac venous drainage [19, 20].

The ME modified bicaval view is the preferred initial window for CS cannulation because it permits complete visualization of cannula insertion and advancement [4–6, 9, 10]. Inclusion of additional windows, in particular the ME4C view, improves CS imaging and success of cannulation [2, 4–7]. Although the length of the CS is not assessed and its ostium is not visualized from the ME2C view, visualization of the CS cannula in this view confirms that the cannula has been advanced to the mid-to distal CS along the posterior and inferior left atrium [4].

Inclusion of the TG-CS view in the authors’ standard comprehensive TEE exam of the CS was prompted by its successful use in a minimally invasive case requiring percutaneous CS cannulation in which none of the ME views successfully imaged the CS. The TG window has previously been described as an option to to image the CS [9]. However, details regarding acquisition and utility of these views are not available. To obtain the TG-CS view, the probe is turned towards the right from the TG LV LAX view with the omniplane angle rotated to 80–100 degrees. Additional advancement of the omniplane angle would enable further visualization of the right heart and vena cavae.

In this study, we found that the CS was visualized in the TG-CS view in 98% of patients. It was visualized in all patients from at least one TEE view, and all four views in 90% of patients. After placement, the CS cannula could be seen in all patients from at least one view.

This retrospective study was limited by its relatively small number of patients. We also did not review our patients’ medical records and therefore may not have captured the subset of patients with variable anatomy and potentially challenging ME or TG coronary sinus imaging. Nevertheless, based on the results reported in this study, the TG-CS view provides an additional modality to image the CS and CS cannula and offers an effective alternative if ME views are limited.

## Conclusion

Retrograde administration of cardioplegia via a CS cannula has become a mainstay of modern cardiac surgery. CS imaging is currently described using a variety of mid-esophageal windows. The TG-CS view offers an additional method of visualizing the CS with a high success rate comparable to ME views. Its inclusion in the assessment of the CS may improve CS imaging and the success of cannulation when ME views are limited.

## Data Availability

All data generated and/or analyzed during this study are included in this published article.

## Abbreviations

CS: Coronary sinus
TEE: Transesophageal echocardiography
ME: Mid-esophageal
ME4C: Mid-esophageal 4-chamber
ME2C: Mid-esophageal 2-chamber
TG: Transgastric
TG-CS: Transgastric coronary sinus
LV: Left ventricle
TG LV LAX: Transgastric left ventricular long axis
